# A High-Reliability 12T SRAM Radiation-Hardened Cell for Aerospace Applications

**DOI:** 10.3390/mi14071305

**Published:** 2023-06-25

**Authors:** Ruxue Yao, Hongliang Lv, Yuming Zhang, Xu Chen, Yutao Zhang, Xingming Liu, Geng Bai

**Affiliations:** 1 Key Laboratory for Wide Band Gap Semiconductor Materials and Devices of Education Ministry, School of Microelectronics, Xidian University, Xi’an 710071, China; yaoruxue4305@126.com (R.Y.); zhangym@xidian.edu.cn (Y.Z.); chenx_1997@163.com (X.C.); ytzhang_xd@163.com (Y.Z.); 2 SMiT Group Fuxin Technology Limited, Shenzhen 518000, China; liuxm@gwxeda.com (X.L.); baigeng@gwxeda.com (G.B.)

**Keywords:** multi-node upset, read stability, single-event effect, static random-access memory (SRAM), write ability

## Abstract

The static random-access memory (SRAM) cells used in the high radiation environment of aerospace have become highly vulnerable to single-event effects (SEE). Therefore, a 12T SRAM-hardened circuit (RHB-12T cell) for the soft error recovery is proposed using the radiation hardening design (RHBD) concept. To verify the performance of the RHB-12T, the proposed cell is simulated by the 28 nm CMOS process and compared with other hardened cells (Quatro-10T, WE-Quatro-12T, RHM-12T, RHD-12T, and RSP-14T). The simulation results show that the RHB-12T cell can recover not only from single-event upset caused by their sensitive nodes but also from single-event multi-node upset caused by their storage node pairs. The proposed cell exhibits 1.14×/1.23×/1.06× shorter read delay than Quatro-10T/WE-Quatro-12T/RSP-14T and 1.31×/1.11×/1.18×/1.37× shorter write delay than WE-Quatro-12T/RHM-12T/RHD-12T/RSP-14T. It also shows 1.35×/1.11×/1.04× higher read stability than Quatro-10T/RHM-12T/RHD-12T and 1.12×/1.04×/1.09× higher write ability than RHM-12T/RHD-12T/RSP-14T. All these improvements are achieved at the cost of a slightly larger area and power consumption.

## 1. Introduction

**Figure 1:**
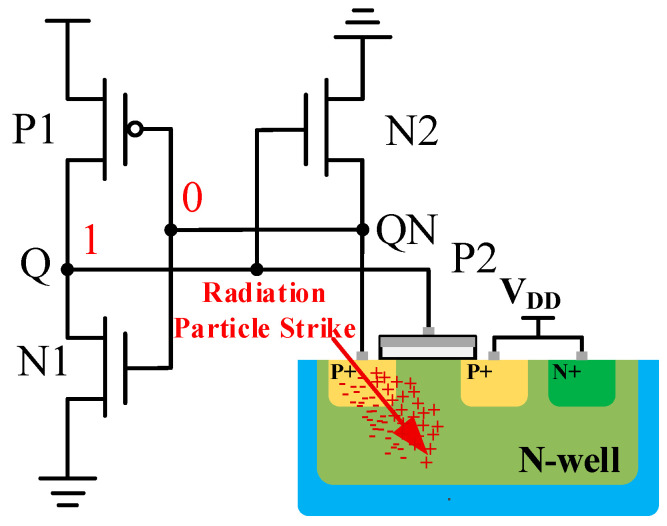
The high-energy particle strikes sensitive nodes in the 6T SRAM inverter pairs.

With the rapid progress of integrated circuit technology, the device feature size and supply voltage gradually decrease, resulting in a continuous decrease in the critical charge of SRAM cells [1,2]. There are a large number of high-energy particles in the space environment, and the running SRAM circuit is highly susceptible to the impact of high-energy particles, which can induce single-event upset (SEU) effects [3]. The storage state is more susceptible to particle striking, causing sensitive node upset and system data access disorder [4]. As shown in Figure 1, suppose that the sensitive point at P2 is bombarded by high-energy particles and that a large number of electron-hole pairs generated are absorbed by this node, generating a transient current forward pulse that raises the voltage of node QN, inducing SEU response and causing a change in the storage data state [5,6]. 

The SRAM cells working in the aerospace environments require high radiation resistance to cope with the complex impact of the environment. Therefore, redundant node reinforcement is often used to harden the design of the cells [7], and the radiation resistance of the cells is improved through redundant feedback mechanisms [8,9]. Over the years, various soft-error-recovery SRAM cells have been proposed [6,10,11,12,13,14,15,16,17,18]. The authors of [10] presented two SRAM-hardened cells, NS10T, and PS10T. However, they enjoy only a partial immunity to SEUs. For example, NS10T can recover from only a “0”→“1” SEU, while PS10T can recover from only a “1”→“0” SEU. DICE cell [11] is a widely known radiation-hardened structure. It has significant overhead in area and power consumption. The use of node separation in the layout can mitigate partial DNUs, but the cell will flip under the tilted incidence of particles. The authors of [12] proposed two soft-error-immune SRAM cells, named QUCCE10T and QUCCE12T. However, QUCCE10T shows a long write delay. Moreover, neither of these cells can recover from a “0”→“1” SEU if sufficient charge is accumulated at the “0”-storing storage node. In addition, some hardened structures have certain soft error recovery effects, such as Nwise-10T [13], SARP-12T [14], QCCS-12T [15], RHMC-12T [16], RH-14T [17], RHS-14T [18], etc. Although the hardened cells have achieved certain radiation tolerance, most structures also suffer from sacrificing area, increasing power consumption, circuit complexity, and reduced stability, failing to achieve a good balance in terms of area, power consumption, read and write time, and radiation tolerance ability. At the same time, as the reduction of the process, the device size and spacing continue to decrease, and single-particle multi-node upset has become a key focus that cannot be ignored in SRAM-hardening design [19,20,21]. The hardened cell needs to have both single-node upset (SNU) and double node upset (DNU) fault tolerance capabilities in order to cope with the impact of complex radiation environments.

In this work, we propose a SRAM-hardened structure called RHB-12T, which has the advantages of the full single-node upset recovery, partial multi-node upset recovery, small footprint, and low power consumption. It meets the requirements of the aerospace environment for both single-node and dual-node upset fault tolerance and low power consumption of SRAM. The next section reviews the standard 6T SRAM and various typical hardened cell structures. The third section introduces the proposed RHB-12T cell and analyzes the normal operation and high robustness to SEUs and SEMNUs of the RHB-12T cell. Then, the fourth section simulates various performance indicators and parameters of the RHB-12T cell using the 28 nm CMOS process and compares and verifies it with typical structures. The fifth section concludes this paper.

## 2. Previous Hardened Memory Cells

**Figure 2:**
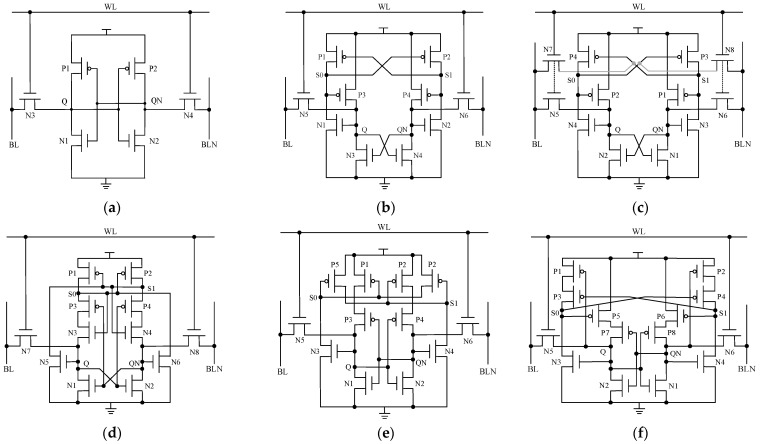
Standard 6T SRAM and classic SRAM radiation-hardening structure: (**a**) 6T SRAM; (**b**) Quattro-10T; (**c**) WE-Quattro-12T; (**d**) RHM-12T; (**e**) RHD-12T; (**f**) RSP-14T.

The standard 6T SRAM is mainly composed of a pair of cross-coupled inverters, as shown in Figure 2a. On account of positive feedback, a change in the value of one storage node triggers the other storage node to change its state, ultimately altering the state of the cell. Therefore, the conventional 6T SRAM fails to meet the requirements of soft-error immunity. Based on the 6T SRAM cell, the researchers designed a variety of radiation-hardening cell structures by using the redundant reinforcement design idea. Through the analysis and understanding of the following classical reinforcement cells, it has a guiding and verifying effect on the new structure.

(1) The Quatro-10T cell has four storage nodes [22], and the circuit structure is shown in Figure 2b. When one of the nodes is affected by radiation, it can be restored to the correct logic value through the feedback of the unaffected node. The use of negative feedback in Quatro-10T can recover from an SEU induced at the “1”-storing storage node but fails to recover from an SEU induced at the “0”-storing storage node. Furthermore, Quatro-10T exhibits poor write ability.

(2) The WE-Quatro-12T consists of 12 transistors [23] and also has four storage nodes inside. The circuit structure is shown in Figure 2c. WE-Quatro-12T is an improved version of Quatro-10T with enhanced writing capabilities. This cell can suppress the influence of the high-energy particles to a certain extent but cannot recover from “0” to “1” SEU. At the same time, the cell has issues with poor read stability and high-power consumption for retention. 

(3) The RHM-12T uses NMOS transistor as the pull-up transistor of the storage node [24], and the sensitive area is small so that the cell can tolerate the voltage disturbance at any sensitive node and can be partially immune to the influence caused by DNU. The circuit structure is shown in Figure 2d. However, due to excessive transistor stacking, its supply voltage scaling is limited. 

(4) The RHD-12T hardened cell can recover from the multi-node upset induced at its internal node-pairs [25]. However, it is incapable of recovering from the SEU induced at the “0” storage node. The circuit structure is shown in Figure 2e. At the same time, the hardened cell also has some disadvantages such as poor data writing capability and high power consumption, which makes it unsuitable for applications in low-power and high-speed environments.

(5) The RSP-14T-hardened cell [26] is optimized in circuit structure and layout based on the structure shown in reference [25], and the circuit structure is shown in Figure 2f. Based on the conventional structure, two PMOS transistors are added to strengthen the redundant nodes and improve the radiation tolerance, and the gate terminal is connected with the storage node, which is beneficial to shortening the time for data writing operation. This structure contains more transistors, which makes the overall area and power consumption of the cell circuit larger.

## 3. The Proposed RHB-12T Cell and Its Operation

### 3.1. Cell Design and Its Operation

**Figure 3:**
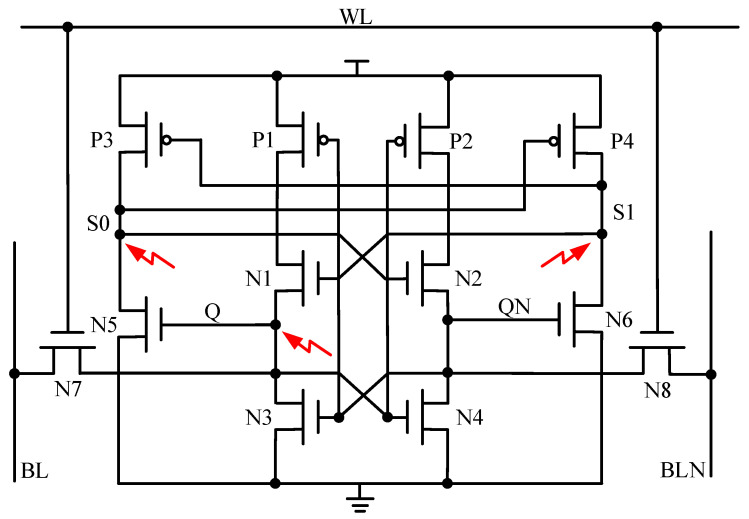
RHB-12T SRAM radiation-hardening cell circuit structure.

**Figure 4:**
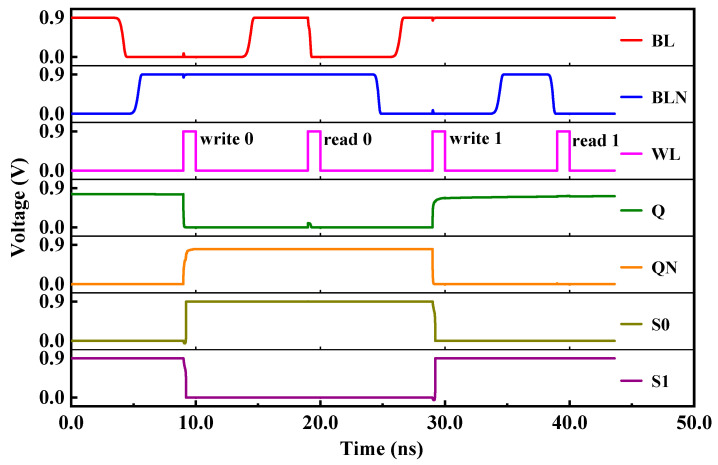
RHB-12T SRAM cell working sequence diagram.

The RHB-12T cell proposed in this paper consists of four PMOS transistors and eight NMOS transistors. The circuit structure of the cell is shown in Figure 3, the red arrows point to the 3 single-event upset sensitive nodes. NMOS transistors N3, N4, N5, and N6 form a pull-down structure, PMOS transistors P1, P2, P3, and P4 form a pull-up structure, and N7 and N8 are access transistors. The cell has four data nodes, two data storage nodes (Q, QN), and two redundant nodes (S0, S1). The nodes Q and QN are connected with BL and BLN under the control of left and right access transistors. The turn-on and turn-off of the access transistor is determined by the word line WL. When WL is at high level, two NMOS access transistors are turned on, and the memory cell reads and writes data. When WL is at low level, the two NMOS access transistors are turned off, and the memory cell latches data. Redundant nodes S0 and S1 enable the hardened cell to recover to the original logic state through the internal feedback mechanism when it is affected by the single-event effect, thus improving the anti-radiation reliability of the cell circuit. The operation sequence of the hardening circuit is shown in Figure 4.

(1) Hold Mode: BL and BLN are both high level, and WL is low level. The two access transistors N7 and N8 are in the off state, which isolates the cell from the bit line and the reverse bit line. A bistable structure is formed inside RHB-12T, and the opposite logic levels are stored at nodes Q and QN. The RHB-12T cell is in the hold mode, and the original storage logic state is not changed without reading and accessing data.

(2) Read Operation: Both BL and BLN are high, and the WL level is set to high. If the stored data is “1”. N7 and N8 are turned on, and node Q is connected to BL through N7, keeping the high level unchanged; node QN is connected with BLN through N8, forming a loop of BLN-N8-N4-GND to discharge, and the voltage of BLN gradually decreases. When a certain voltage difference is formed between BL and BLN, the stored logic value “1” can be read out by the sense amplifier of the peripheral circuit.

(3) Write Operation: WL is high level, and BL and BLN are set to a high level and a low level to realize the write operation. Assuming that the original stored data is “1” and the pre-write logic is “0”, set the BL level to low and the BLN level to high. The WL level is high, N7 and N8 are in the conduction state, node Q is connected to BL through N7 and pulled to “0” state, and node QN is charged to “1” state by BLN through N8. At this time, the storage logic of the RHB-12T cell is reversed, and the node Q point becomes “0” and the QN point becomes “1”, thus completing the cell writing operation.

### 3.2. SEU Recovery Analysis

**Figure 5:**
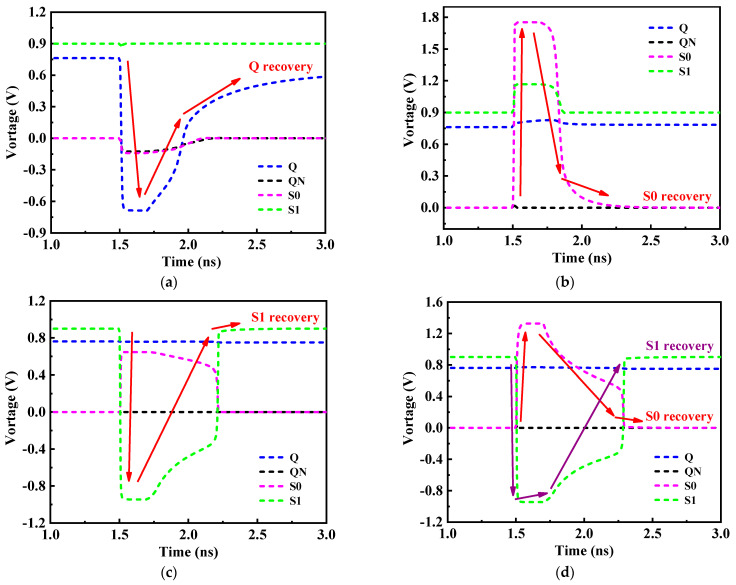
Response of different nodes of the RHB-12T SRAM-hardened cell when struck by high-energy particles: (**a**) node Q; (**b**) node S0; (**c**) node S1; (**d**) node S0 and S1.

When the drain of PMOS transistor is subjected to the high-energy particles, only an upward transient current (“1→1” or “0→1”) may be formed in the sensitive region. However, when the drain terminal of NMOS transistor is subjected to the high-energy particles, a downward transient current (“1→0” or “0→0”) is generated [27]. If the original state of RHB-12T is high logic, the nodes Q = 1, QN = 0, S0 = 0, and S1 = 1. The node QN storing “0” state is surrounded by NMOS transistor, which can only generate “0→0” pulse, and the logic level of stored data is not upset, so QN is not a sensitive node. Therefore, when the storage logic of the hardened cell is high, there are only three sensitive nodes in the circuit: Q, S0, and S1. The fault-tolerant process of the RHB-12T cell under the conditions of single-node upset and double-node upset is analyzed in detail, and the recovery of each node is shown in Figure 5.

(1) SEU at Q: When node Q is affected by SEU, its logical state changes from “1” to “0”. The logic changed at node Q causes transistors N4 and N5 to turn off and transistor P2 to turn on. At the same time, both N4 and N5 are turned off, so that the Q node is completely isolated from the QN and S0 nodes, meaning that the QN and S0 nodes both remain unchanged (QN = 0, S0 = 0). The QN node is “0” to turn on the transistor P1; the node S0 is at a low level, so that the transistor P4 is turned on; the node S1 maintains a high logic, which turns transistor N1 on. At this time, the transistors P1 and N1 are both turned on, while N3 is turned off, and the point Q is charged by VDD through P1 and N1, and the point Q is restored to “1”, thus realizing the single-node self-recovery process at the node Q (Figure 5a).

(2) SEU at S0: When the S0 node is struck by high-energy particles, the logic state of node S0 changes from “0” to “1”, which makes transistor P4 turn into temporary off and transistor N2 turn into temporary on. Analysis of the remaining nodes of the hardened cell shows that the data upset phenomenon of S0 node does not affect the other three data nodes. Therefore, the remaining nodes of the hardened cell keep the original logical state unchanged, that is, Q = 1, QN = 0, and S1 = 1. Transistor N5 is in the on state because node Q is at the high level. Transistor P3 is in the off state, because node S1 is at the high level, and node S0 is pulled down and restored to the original “0” state. Node S0 realizes the single-node self-recovery process of this node (Figure 5b).

(3) SEU at S1: When the S1 node is struck by high-energy particles, the S1 node changes from “1” to “0”, which leads to transistor P3 conduction, and VDD charges the node S0 to “1” through P3. Therefore, the node S0 temporarily changes from “0” to “1”. However, node Q is not affected by the changes of the logic states of nodes S0 and S1 and maintains the original “1” state unchanged. Node Q = 1, so that transistor N5 is on, and node S0 is connected to GND through transistor N5 and pulled down to “0”. Similarly, node Q = 1 generates N4 in the on state, node QN keeps the original “0” unchanged, and N6 is in the off state; Node S0 is pulled back to low level again, and point S0 becomes low logic again, so that P4 becomes conductive. In this case, P4 is turned on and N6 is turned off, so that node S1 is pulled up to the original “1” state by VDD, and the single-node self-recovery process of node S1 is realized Due to the increase in feedback action time, the recovery time of the S1 node is greater than that of the S0 node (Figure 5c).

(4) SEU at S0-S1: When nodes S0 and S1 are struck by high-energy particles, the logical states of the nodes S0 and S1 are upset, so the node S0 changes from “0” to “1” and the node S1 changes from “1” to “0”. At this time, N2 and P3 are temporarily turned on, and P4 is turned off. The storage logic changes of the nodes S0 and S1 does not affect the nodes Q and QN, whose original logical states remain unchanged. Node Q = 1, and N5 is turned on so that node S0 is pulled down to a low level and restored to the original “0” state. While node S0 = 0, P4 becomes conductive again, which makes node S1 pull up to high logic and restore to the original “1” logic. The self-recovery process of {S0, S1} double-node upset is successfully realized at nodes S0 and S1 (Figure 5d).

**Figure 6:**
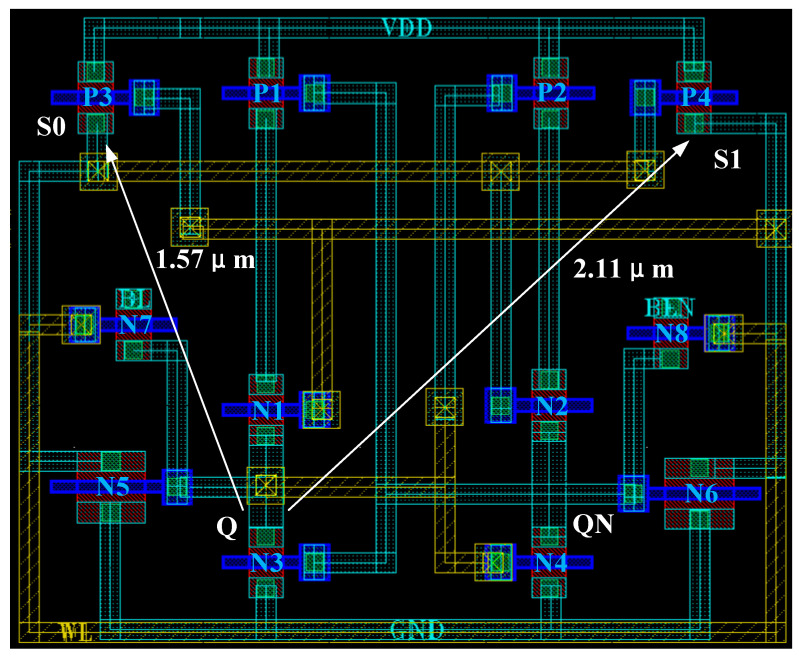
RHB-12T radiation-hardened cell layout design.

Therefore, the RHB-12T-hardened cell has the ability of single-node Q (or QN), S0, S1, and double-node {S0, S1} to upset and recover and can completely suppress SNU and partially tolerate DNU response. The research shows that the charge-sharing effect between two NMOS (or PMOS-NMOS) occurs at 1.62 μm (or 0.6 μm) [28,29]. The physical distance between node Q and nodes S0 and S1 is reasonably designed on the layout to effectively reduce the influence of the charge-sharing effect. Figure 6 shows the layout of the RHB-12T-hardened cell. The physical distances between the cell node Q and the nodes S0 and S1 are set to be greater than the effective distances of PMOS and NMOS (S0-Q and S1-Q are 1.57 μm and 2.11 μm, respectively), which effectively reduces the possibility of {Q, S0} and {Q, S1} double-node upset caused by the charge-sharing effect and improves the SEU tolerance of the cell.

## 4. Simulation and Analysis

In this section, based on Cadence circuit simulation software, the RHB-12T radiation-hardened cell is designed and simulated by the TSMC 28 nm CMOS process, which verifies the performance of the hardened cell in terms of reading and writing speed, stability, power consumption, and radiation tolerance and provides data support for the subsequent performance comparison between the RHB-12T cell and other classic structures.

### 4.1. Access Time Simulation

**Figure 7:**
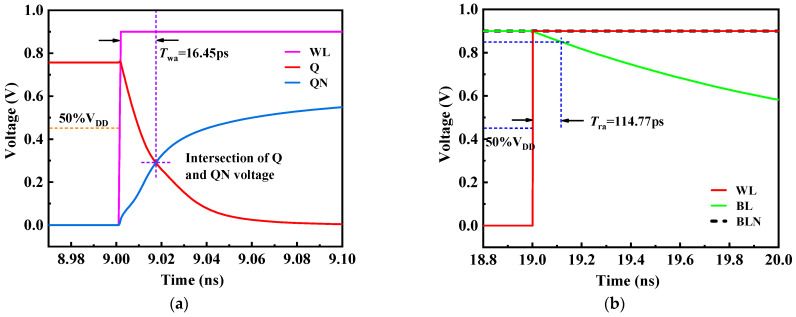
RHB-12T cell access time simulation: (**a**) write time simulation; (**b**) read time simulation.

Access time is an important index to measure the working speed of SRAM memory, including writing time and reading time. As shown in Figure 7a, which is the simulation result of the write time of the RHB-12T cell, the time difference between the moment when the word line WL rises to 0.5× VDD and the moment when nodes Q and QN intersect is defined as the write time of the memory cell. The simulation results show that the time *Twa* of writing the data to be written into the storage node by the RHB-12T cell is 16.45 ps. Similarly, the read time is defined as the time interval between the time when the word line WL rises to 0.5× VDD and the time when the bit line BL falls by 50 mV. As shown in Figure 7b, the time *Tra* for the RHB-12T cell to read data from the storage node is 114.77 ps.

### 4.2. Stability Simulation

**Figure 8:**
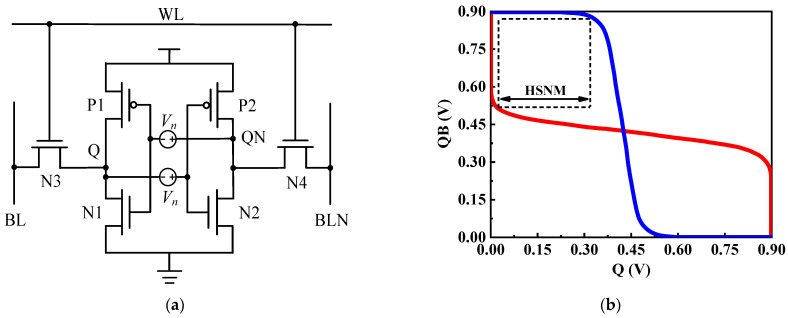
SNM simulation: (**a**) simulation circuit diagram; (**b**) butterfly curve diagram.

The static noise margin (SNM) is an important parameter to measure the stability of the cell, which is defined as is the maximum DC noise voltage that the memory cell can tolerate when working. The larger the SNM value, the stronger the anti-interference ability of the circuit and the more stable the circuit is. According to the different working modes of SRAM, SNM can be divided into three types: read static noise tolerance (RSNM), write static noise tolerance (WSNM), and keep static noise tolerance (HSNM). Figure 8 shows the equivalent diagram and butterfly diagram of the SNM simulation test of the memory cell. The linear DC characteristics of the storage node are simulated by adding a voltage noise source Vn to the input of the cross-coupled inverter of the SRAM memory cell, and the two voltage transmission curves obtained are superimposed to form a butterfly diagram. The side length of the largest inscribed square in the butterfly diagram is the static noise tolerance. In this paper, the butterfly curve analysis method is used to measure the static noise tolerance of the RHB-12T cell in reading operation, writing operation, and holding operation, and three kinds of SNM data are shown in Table 1.

### 4.3. Cell Power Consumption Simulation

With the increasing process integration and working performance requirements, power consumption has become an important parameter to measure the excellent performance. The power consumption of the SRAM cell circuit is divided into two types: static power consumption and dynamic power consumption.

(1) Static power consumption: the power consumption in the data holding stage is caused by the reverse leakage current. The expression for calculating the static power consumption caused by the leakage current is

(1) $$P_{leak}=\;\;V_{DD}\times \;I_{leak}$$

where *V_DD_* is the supply voltage and *I_leak_* is the leakage current. The power supply voltage is 0.9 V, and the leakage current of the RHB-12T cell is 6.52 nA so that the static power consumption is 5.87 nW.

(2) Dynamic power consumption: Switching power consumption of charging and discharging the load capacitor and short-circuit power consumption caused by instantaneous simultaneous conduction of the circuit MOSFET transistor. Therefore, the dynamic power consumption is the sum of the two power consumptions, which is expressed as follows:

(2) $$\begin{array}{l} P_{dyamic}=\;\;P_{switching}+\;\;P_{short}\\ \;\;\;\;\;\;\;\;\;=\;2\times C_{load}\times V_{DD}^{2}\times f_{clock}+V_{DD}\times I_{short} \end{array}$$

where *P_dyamic_* is the dynamic power consumption, *P_switching_* is the switching power consumption, *P_short_* is the short-circuit power consumption, *C_load_* is load capacitance, *V_DD_* is the power supply voltage, *f_clock_* is the clock frequency, and *I_short_* is the short-circuit current. Under the simulation condition that the power supply voltage is 0.9 V, the dynamic power consumption of the RHB-12T hardened cell is 24.09 nW.

### 4.4. Simulation of Radiation Tolerance

In this section, the pulse current source will be used to simulate the circuit-level single-event effect of the RHB-12T-hardened cell to verify the performance of the radiation tolerance characteristics of the proposed RHB-12T cell.

(1) Pulse current model

In order to verify the radiation ability of the proposed RHB-12T cell, the double exponential current model is used to simulate the influence of high-energy particles [30]. Injecting the current into the sensitive node of the circuit generates a positive transient pulse at the drain of PMOS and a negative transient pulse at the drain of NMOS. The injection current is expressed with the following formula:

(3) $$\left\{\begin{array}{l} I(t)=I_{o}\left(e^{-\frac{t}{\alpha }}-e^{-\frac{t}{\beta }}\right)\\ I_{o}=\frac{Q_{\mathrm{tot}}}{\alpha -\beta } \end{array}\right.$$

where $I_{o}$ is approximated as the peak current, $Q_{tot}$ is the total deposited charge, α stands for the collection time constant of the junction, and β represent the time constant for initial ion track establishment.

(2) Node-injection verification

**Figure 9:**
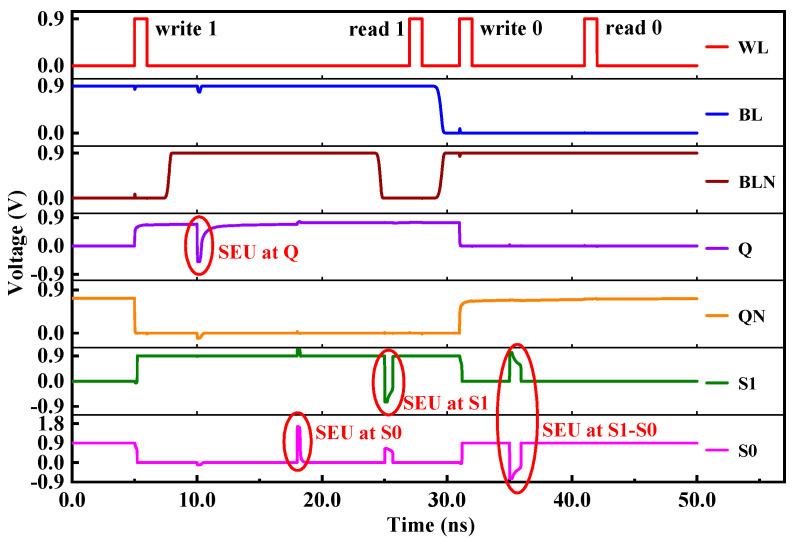
Single-event effect simulation of RHB-12T-hardened cell.

There are three sensitive nodes, Q, S0 and S1, in the storage cell of RHB-12T. In order to verify the self-recovery performance of the sensitive nodes of the hardened cell after being struck by high-energy particles, the transient current source model of the single-event effect is used to characterize the response of the sensitive nodes to high-energy particles. The simulation results of node recovery are shown in Figure 9. The simulation results show that both Q, S0 and S1 sensitive single nodes and {S0, S1} double nodes of the RHB-12T storage cell can recover from the upset to the original logic level. The recovery process is consistent with the fault-tolerant analysis in the above 4.2 summary, and the cell has the ability of self-recovery from the upset of single nodes Q, S0, and S1 and double nodes {S0, S1}.

(3) Critical charge simulation

**Figure 10:**
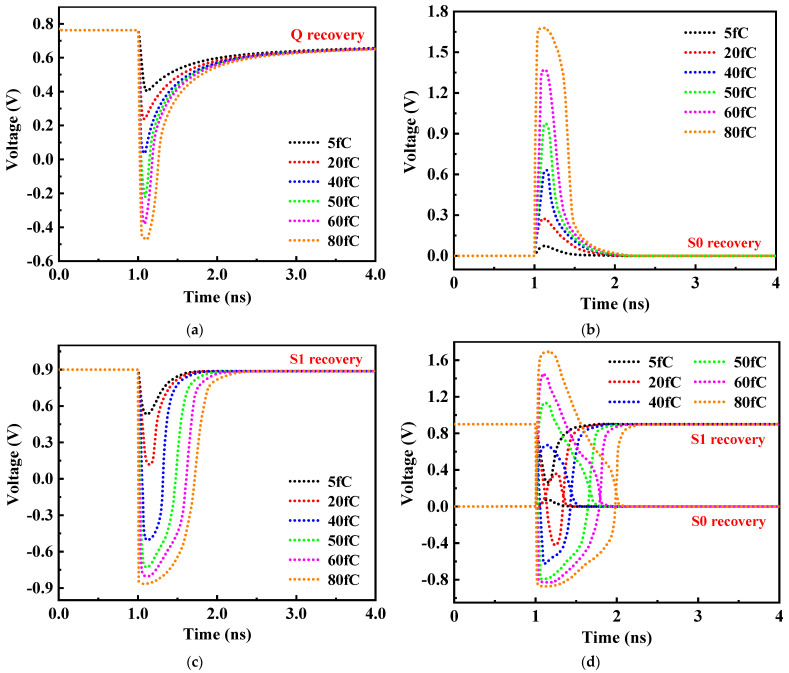
RHB-12T SRAM-hardened cell collects different amounts of charge at different nodes in response to (**a**) node Q; (**b**) node S0; (**c**) node S1; and (**d**) nodes S0 and S1.

Critical charge refers to the minimum charge collected by sensitive nodes that can change the original stored data, and it is a parameter index to measure the fault tolerance of memory cells. Figure 10 shows the response of different sensitive nodes of the RHB-12T-hardened cell to collecting different amounts of charges. The simulation results show that when 80 fC charges are collected on the sensitive nodes Q, S0, S1 and the double nodes {Q, S0} of the RHB-12T cell, the nodes can still recover to the initial logic state after being upset by high-energy particles. At the same time, with the increasing number of charges injected by sensitive nodes, the degree of node upset becomes more intense and the characterization is more seriously affected by a single-event effect. After repeated pulse injection of three sensitive nodes and {Q, S0} double nodes, it is found that each sensitive node of the RHB-12T-hardened cell still has recovery ability when the charge is injected at 200 fC.

### 4.5. Cell Performance Summary

In order to measure the comprehensive performance of the proposed new RHB-12T-hardened cell, this work compares the performance of the RHB-12T-hardened cell with various typical SRAM anti-radiation hardening structures. To ensure fairness in performance comparison, all cells were designed using the same 28 nm CMOS process and the minimum size design was used for all size settings except for necessary transistors. At the same time, all cells were ensured to be in a simulation environment with consistent timing settings and peripheral circuits.

(1) This work compares the radiation tolerance performance of the proposed RHB-12T-hardened cell with various other typical SRAM-hardened structures, and the comparison results are shown in Table 2. Compared to the hardened structures of Quatro-10T, WE-Quatro-12T, RHD-12T, and RSP-14T, which have four sensitive nodes, the RHB-12T structure proposed in this paper only has three sensitive nodes. The reduction in the number of sensitive nodes reduces the possibility of single-event effects occurring when the cell is struck by high-energy particles. Meanwhile, compared to some typical SRAM-hardened cells that are tolerant to single-node upset, RHB-12T-hardened cells not only have complete tolerance for arbitrary upset of all sensitive single nodes but also have the ability to self-recover from double node {S0, S1} upset. The RHB-12T cell proposed in this work can still recover to its original logical state after upsetting when collecting 200 fC charges at sensitive nodes. Compared with other SRAM-hardened structures, the RHB-12T cell has the highest critical charge value and strong radiation tolerance to withstand the influence of higher injected high-energy particles.

(2) Table 3 shows the results of the performance comparison data between the RHB-12T-hardened cell and other typical SRAM-hardened cell structures. The proposed RHB-12T cell is DNU resilient, and it has a similar reading and writing time to the unhardened 6T cell. The proposed cell exhibits 1.14×/1.23×/1.06× shorter read delay than Quatro-10T/WE-Quatro-12T/RSP-14T and 1.31×/1.11×/1.18×/1.37× shorter write delay than WE-Quatro-12T/RHM-12T/RHD-12T/RSP-14T. Compared to previous radiation-hardened memory cells, RHB-12T has both large RSNM and WSNM. The proposed cell exhibits 1.35×/1.11×/1.04× higher read stability than Quatro-10T/RHM-12T/RHD-12T and 1.12×/1.04×/1.09× higher write ability than RHM-12T/RHD-12T/RSP-14T. However, the improvement of the circuit structure may also bring some defects. Firstly, the increase in additional transistors will inevitably lead to an increase in area. Secondly, as a pull-up transistor, NMOS can cause threshold loss during high-level transmission, resulting in weak high-level storage logic that is susceptible to external noise interference while in a hold state.

## 5. Conclusions

This work proposes an RHB-12T-hardened cell structure for the soft-error recovery, which has the ability of the single-node and partial multi-node upset self-recovery. The simulation results based on the 28 nm CMOS process show that the RHB-12T-hardened cell achieves a good balance between speed, area, stability, and reliability. The proposed cell exhibits 1.14×/1.23×/1.06× shorter read delay than Quatro-10T/WE-Quatro-12T/RSP-14T and 1.31×/1.11×/1.18×/1.37× shorter write delay than WE-Quatro-12T/RHM-12T/RHD-12T/RSP-14T. It also shows 1.35×/1.11×/1.04× higher read stability than Quatro-10T/ RHM-12T/RHD-12T and 1.12×/1.04×/1.09× higher write ability than RHM-12T/RHD-12T/RSP-14T. However, the improvement of this structure also brings certain drawbacks. Firstly, the increase in the redundant transistors will inevitably increase the cell area. Secondly, as a pull-up transistor, NMOS causes threshold loss during high-level transmission, resulting in weak high-level storage logic that is susceptible to external noise interference while in a holding state

At present, researchers have proposed some effective hardened schemes, which, to some extent, inhibit the effect of the SEU. However, while SRAM cells have high radiation resistance, lower and smaller power consumption, area, and storage time are still research difficulties and hotspots. Meanwhile, with the rapid development of integrated circuit technology, the size and spacing of devices continue to decrease. The phenomenon of single-event multi-bit upsets and single-event multi-cell upsets will also become a difficult point that cannot be ignored in SRAM circuit-hardened design in the future.

## Figures and Tables

**Table 1:** Three static noise tolerance data sorting results of RHB-12T-hardened cell.

RSNM/mV	WSNM/mV	HSNM/mV
175.72	315.42	286.87

**Table 2:** Comparison of anti-radiation performance between RHB-12T-hardened cell and other SRAM-hardened cell structures.

Structure Name	Number of Sensitive Nodes	Tolerance SEU?	Tolerance Type	Tolerance DNU?	Critical Charge/fC
STD-6T	2	No	-	No	-
Quatro-10T [22]	4	No	1→0	No	25.30
WE-Quatro-12T [23]	4	No	1→0	No	35.47
RHM-12T [24]	3	Yes	1→0, 0→1	Yes	87.65
RHD-12T [25]	4	No	1→0	Yes	112.37
RSP-14T [26]	4	Yes	1→0, 0→1	Yes	140.45
This Work	3	Yes	1→0, 0→1	Yes	>200

**Table 3:** Performance comparison data results between RHB-12T-hardened cell and other typical SRAM-hardened cell structures.

Structure Name	Static Power /nW	Dynamic Power /μW	Area /μm^2^	Write Time /ps	Read Time /ps	RSNW /mV	WSNW /mV	HSNW /mV
STD-6T	5.83	22.42	0.58	10.32	90.68	100.4	215.92	178.39
Quatro-10T [22]	8.29	38.45	0.94	32.87	130.52	135.29	363.01	310.32
WE-Quatro-12T [23]	10.97	23.56	1.08	21.56	140.77	209.30	346.37	293.41
RHM-12T [24]	5.69	27.14	1.02	18.21	115.69	158.61	281.45	246.42
RHD-12T [25]	6.19	24.35	0.98	19.35	117.74	169.54	303.43	301.47
RSP-14T [26]	5.05	23.25	1.22	22.54	121.63	177.11	290.09	250.13
This work	5.89	24.09	0.96	16.45	114.77	175.72	315.42	286.87

## Data Availability

Not applicable.
